# Chemotherapy-Induced Infertility: Mechanisms, Risk Factors, and Fertility Preservation Strate-gies in Cancer Patients

**DOI:** 10.5935/1518-0557.20260014

**Published:** 2026

**Authors:** Amirreza Talaie, Sanaz Alaee, Soudabeh Sabetian, Fatemeh Zal, Amin Tamadon

**Affiliations:** 1 Plant Design Department, Pars Abnoos Sanat Consulting Engineering Company, Shiraz, Iran; 2 Department of Reproductive Biology, School of Advanced Medical Sciences and Technologies, Shiraz University of Medical Sciences, Shiraz, Iran; 3 Stem Cells Technology Research Center, Shiraz University of Medical Sciences, Shiraz, Iran; 4 Infertility Research Center, Shiraz University of Medical Sciences, Shiraz, Iran; 5 Department of Biochemistry, School of Medicine, Shiraz University of Medical Sciences, Shiraz, Iran; 6 Department of Natural Sciences, West Kazakhstan Marat Ospanov Medical University, Aktobe 030012, Kazakhstan

**Keywords:** chemotherapy, infertility, fertility preservation, gonadotoxicity, reproductive health

## Abstract

Chemotherapy remains a cornerstone of cancer treatment, effectively targeting rapidly dividing cancer cells, yet its systemic effects often extend to healthy reproductive cells, resulting in temporary or permanent infertility. This review explores the mechanisms by which chemother-apy impairs fertility in both men and women, focusing on the damage to ovarian follicles and spermatogenesis. Key factors influencing fertility outcomes include the type and dosage of chemotherapy drugs, patient age, and pre-existing reproductive health. Alkylating agents, such as cyclophosphamide, and platinum-based drugs, like cisplatin, pose the highest risk of gonad-otoxicity, while other agents exhibit milder, often reversible effects. Fertility preservation op-tions such as oocyte and embryo freezing for women, sperm banking for men, and emerging techniques like ovarian and testicular tissue cryopreservation offer hope for maintaining re-productive potential. The psychological burden of infertility and the promising yet complex role of nanoparticles in reproductive medicine are also discussed. By synthesizing current research, this review underscores the need for personalized fertility counseling and advanced preserva-tion strategies to balance cancer treatment efficacy with reproductive goals.

**Figure:**
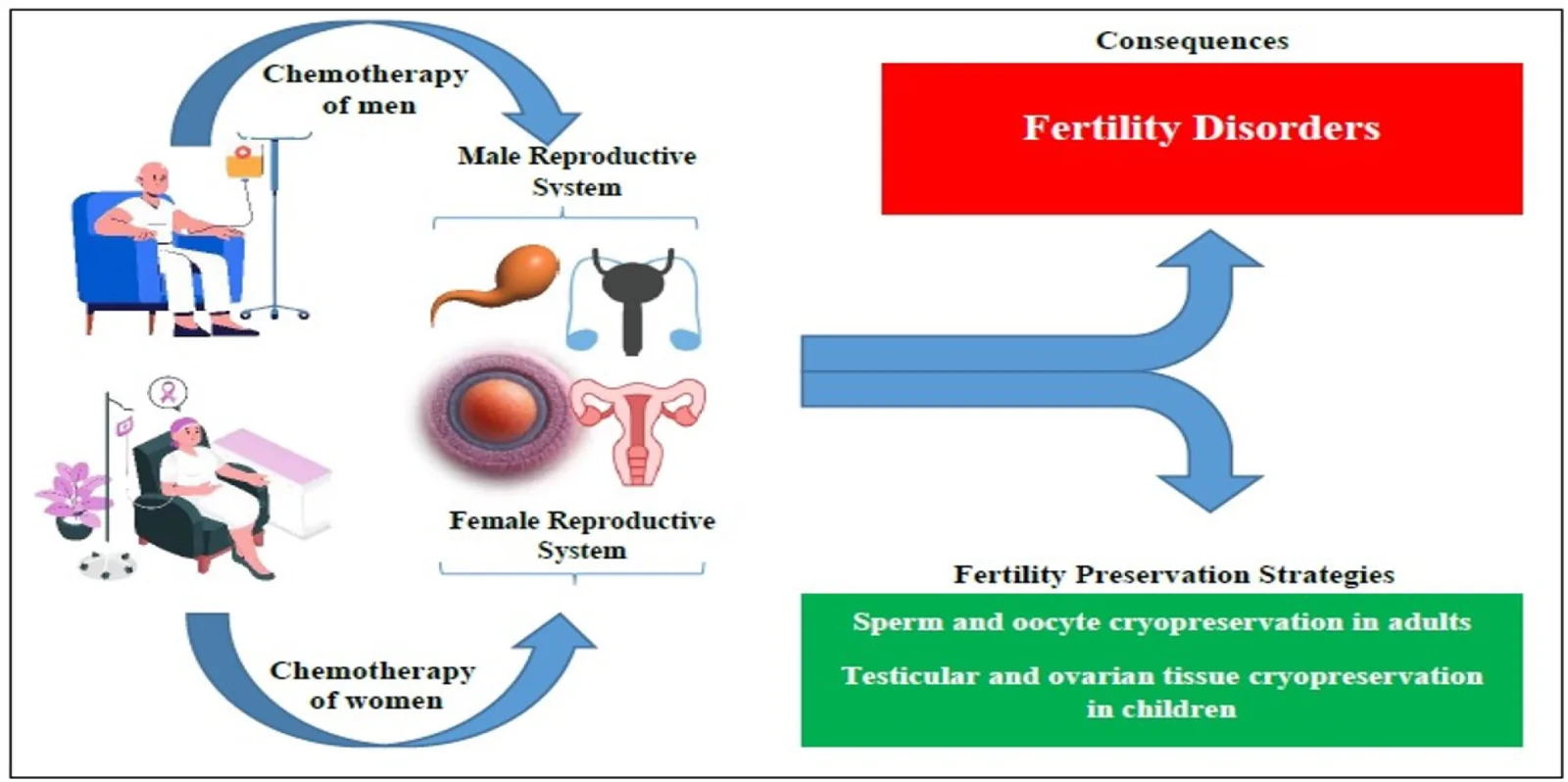

## INTRODUCTION

Chemotherapy is a potent and systemic treatment used to target rapidly dividing cancer cells, but it can inadvertently affect healthy cells in the body, such as cells in the reproductive system (Mustapha *et al.*, 2021). Both men and women have reproductive cells that are sensitive to chemotherapy, and the damage caused by these drugs can lead to temporary or permanent infertility. This unintended consequence is due to chemotherapy’s mechanism of action, which targets not just cancer cells but any other cells, including those responsible for oocyte and sperm production (Papathanasiou *et al.*, 2024). The risk of infertility depends on several factors, including the specific drugs used, the dosage, the patient’s age, and their pre-existing fertility health (Papathanasiou *et al.*, 2024).

In women, chemotherapy primarily affects the ovaries, which contain a finite number of oocytes from birth (Papathanasiou *et al.*, 2024). These oocytes do not regenerate, so any damage to them is permanent. Chemotherapy drugs, particularly alkylating agents such as cyclophosphamide, can cause significant ovarian toxicity by damaging the DNA in oocytes or triggering cell death (Markowska *et al.*, 2024). As a result, chemotherapy can reduce the ovarian reserve, leading to diminished fertility or even premature ovarian failure (POF), which is an early onset of menopause (Horan, 2022). Women undergoing chemotherapy may experience temporary amenorrhea, but in some cases, this can become permanent. The extent of damage is often age-dependent; younger women tend to have a better chance of recovering fertility after treatment, whereas older women, who have fewer oocytes, are at a greater risk of permanent infertility (Horan, 2022; Sabetian *et al.*, 2022a).

In men, chemotherapy can severely impact spermatogenesis, the process of sperm production in the testes (Delessard *et al.*, 2020; Yaghutian Nezhad *et al.*, 2021). Spermatogonia are rapidly dividing and thus vulnerable to chemotherapy’s effects. Chemotherapy can reduce or disrupt sperm production, leading to conditions like oligospermia or azoospermia (Javed, 2020). Depending on the type of drug and dosage, this infertility may be temporary. Even if sperm production resumes, chemotherapy can cause genetic damage to the DNA within sperm, which raises concerns about the health of future offspring (Meistrich, 2020). As with women, the age of the patient plays a significant role; younger men are more likely to recover sperm production after treatment, while older men may face a higher risk of irreversible infertility (Jensen *et al.*, 2022).

The type of chemotherapy drug used plays a significant role in determining fertility outcomes. Alkylating agents, such as cyclophosphamide and busulfan, are particularly damaging to reproductive cells (Bhardwaj *et al.*, 2023; Bahmyari *et al.*, 2024). Platinum-based drugs, like cisplatin, are also highly gonadotoxic and can cause severe ovarian or testicular damage (Tian En *et al.*, 2020). Other chemotherapy drugs, such as taxanes and anthracyclines, pose a moderate risk to fertility but may still cause significant reproductive harm, especially when used in high doses or combined with other treatments (Poorvu *et al.*, 2019). The total dose and length of chemotherapy also influence the likelihood of infertility, with higher doses and longer treatments posing a greater risk (Hales *et al.*, 2005; Markowska *et al.*, 2024).

Ultimately, the effects of chemotherapy on fertility can vary greatly depending on individual circumstances. Age, the type of cancer, the specific chemotherapy drugs, and the patient’s overall reproductive health all play a role in determining whether fertility will be affected temporarily or permanently (Li *et al.*, 2024). While chemotherapy is a crucial tool in the fight against cancer, its potential to cause infertility can have profound psychological and emotional impacts on patients, especially those of reproductive age (Goldman *et al.*, 2015). Therefore, patients need to discuss their fertility concerns with their healthcare providers before beginning treatment to explore possible preservation options.

Table 1 provides a comprehensive snapshot of the fertility effects associated with twelve widely used chemotherapy drugs-Cyclophosphamide, Doxorubicin, Paclitaxel, Cisplatin, Methotrexate, Fluorouracil (5-FU), Vincristine, Etoposide, Bortezomib, Carboplatin, Ifosfamide, Bleomycin, and Gemcitabine-alongside Bumetanide, a non-chemotherapy diuretic, as of February 27, 2025. The data reveals a spectrum of reproductive impacts, ranging from severe, often permanent infertility risks posed by alkylating agents like Cyclophosphamide and Ifosfamide, to milder, potentially reversible effects from drugs like Gemcitabine and Bleomycin. Platinum-based agents such as Cisplatin and Carboplatin show significant but varying degrees of gonadotoxicity, while targeted therapies like Bortezomib and less toxic agents like Vincristine and Fluorouracil demonstrate minimal direct impact on fertility (Abdel-Latif *et al.*, 2022; American Cancer Society and American Society of Clinical Oncology (ASCO), 2025). Bumetanide stands apart with no evidence of fertility effects, underscoring its distinct role outside oncology (NHS - National Health Service, 2022). High-risk drugs necessitate proactive fertility preservation strategies, such as oocyte or sperm banking, particularly for younger patients, while those with milder profiles may allow for natural recovery post-treatment (Li *et al.*, 2024). However, gaps in research-especially for newer agents like Bortezomib and less-studied drugs like Gemcitabine-suggest a need for further investigation to fully elucidate their long-term effects, particularly in women. This table serves as a valuable resource for clinicians and patients navigating treatment decisions, emphasizing the importance of individualized counseling to balance cancer management with reproductive goals.

**Table 1:** Effects of chemotherapy drugs and bumetanide on fertility: a comprehensive overview of commonly used agents and their reproductive impacts.

Drug Name	Effects on Fertility	References
Cyclophosphamide	It can cause infertility, particularly in women, leading to premature ovarian failure. Risk increases with age and dose. In men, it may cause azoospermia or oligospermia. Fertility preservation (e.g., oocyte/sperm freezing) is recommended.	Huong *et al.*, 2002; Gajjar *et al.*, 2015; Mansilla *et al.*, 2019
Doxorubicin	It may cause ovarian toxicity in women, reducing ovarian size and ovulation rates, potentially leading to infertility. Limited data on male fertility, but some gonadal impact is possible.	Ben-Aharon *et al.*, 2010; Poorvu *et al.*, 2019; Mohan *et al.*, 2021
Paclitaxel	Likely has transient effects on fertility, with milder ovarian toxicity in women and potential recovery. In men, it may cause temporary sperm DNA damage, but the effects are less severe than other drugs.	Tarumi *et al.*, 2009; Ili *et al.*, 2019; Ma *et al.*, 2020
Cisplatin	It can cause significant infertility, especially in men, with reduced sperm production and testicular damage. In women, it affects ovarian reserve, leading to potential infertility.	Schrader *et al.*, 2001; Abdel-Latif *et al.*, 2022; ASCO, 2025
Methotrexate	It may have temporary effects on fertility, particularly in women, with reversible impacts on oocyte production. In men, recent studies suggest minimal long-term fertility impact.	French *et al.*, 2003; Zarén *et al.*, 2023
Fluorouracil (5-FU)	Generally has minimal direct impact on fertility, with limited evidence of significant gonadal toxicity. Effects may be secondary to overall treatment regimen rather than the drug alone.	Fuchs *et al.*, 2017; Razzaque, 2018; NCI, 2025
Vincristine	Typically has low gonadotoxicity, with minimal direct effects on fertility in both men and women. Recovery of reproductive function is common post-treatment.	Bandino *et al.*, 2018; NIDDK, 2023
Etoposide	Can impair fertility, particularly in men, with potential for azoospermia at high doses. In women, it may reduce ovarian reserve, though effects are less severe than alkylating agents.	Cavalcanti *et al.*, 2006; Joyisa *et al.*, 2019
Bumetanide	No direct evidence suggests it affects fertility in men or women. As a diuretic, not a chemotherapy drug, it lacks gonadotoxic properties typical of cancer treatments.	NHS, 2022; Drugs.com, 2025
Bortezomib	Limited evidence suggests minimal direct impact on fertility. Studies indicate it may not significantly affect ovarian or testicular function, though data is sparse and often confounded by combination therapies.	Zhen *et al.*, 2018; NIDDK, 2020
Carboplatin	Can affect fertility, particularly in men, with potential for reduced sperm count, though less severe than Cisplatin. In women, it may cause ovarian damage, but the risk of permanent infertility is lower than with Cisplatin.	Razzaque, 2018; Joyisa *et al.*, 2019; NCI, 2024
Ifosfamide	Another alkylating agent with significant infertility risks, similar to Cyclophosphamide. Can cause premature ovarian insufficiency in women and azoospermia in men, with dose-dependent effects. Fertility preservation is often advised.	Huang *et al.*, 2005; Fuchs *et al.*, 2017
Bleomycin	Used in testicular cancer, with moderate fertility effects, often reversible in men. In women, ovarian impact is less studied but appears minimal compared to alkylating agents.	ASCO, 2025
Gemcitabine	An antimetabolite with limited fertility data but potentially milder effects. It may cause temporary ovarian or testicular dysfunction, with evidence suggesting recovery in many cases.	Razzaque, 2018; Joyisa *et al.*, 2019; ASCO, 2025

Recent updates in clinical practice guidelines from the American Society of Clinical Oncology underscore the importance of discussing and offering fertility preservation options to all patients of reproductive age at the time of cancer diagnosis (Su *et al.*, 2025). All guidelines endorse established methods such as embryo, oocyte, and ovarian tissue cryopreservation, as well as ovarian transposition (ESHRE Guideline Group on Female Fertility Preservation *et al.*, 2020; Kieu *et al.*, 2022). For females, options include embryo cryopreservation, oocyte freezing, and ovarian tissue freezing. Hormonal suppression and ovarian shielding during radiation may also help reduce damage. However, these methods often require time-sensitive procedures, which can be challenging when urgent cancer treatment is needed. For males, sperm banking is the most common and effective method, while testicular sperm extraction or testicular tissue freezing may be options for those unable to produce a sample (Ní Dhonnabháin *et al.*, 2022; Pathak *et al.*, 2023; Katzir *et al.*, 2024). The Cancer Council Australia emphasizes the importance of early discussions between patients and healthcare providers to explore fertility preservation before treatment begins. A multidisciplinary approach involving oncologists, fertility specialists, and counselors ensures patients receive comprehensive care, including psychological and financial support (Kieu *et al.*, 2022).

This review integrates such up-to-date guideline recommendations and pooled clinical evidence to provide a robust framework for assessing fertility risks and preservation strategies in men and women undergoing gonadotoxic treatments. Also includes preclinical research using animal models to provide a comprehensive understanding of chemotherapy-induced gonadotoxicity and strategies for fertility preservation.

We searched PubMed, Scopus, Web of Science, and Embase using keywords such as ‘chemotherapy AND fertility preservation,’ ‘gonadotoxicity,’ ‘premature ovarian failure,’ and ‘oocyte cryopreservation.’ The search covered publications from January 2015 to June 2025. Only English-language articles were included.

## CHEMOTHERAPY’S EFFECTS ON FEMALE FERTILITY

Female fertility is intricately tied to the health and number of ovarian follicles containing immature oocytes (Telfer *et al.*, 2023). From birth, a woman has a fixed number of oocytes that are gradually lost throughout her life, with no possibility of regeneration (Moghadam *et al.*, 2022). These oocytes are highly sensitive to environmental factors, including chemotherapy, which can cause both temporary and permanent damage (Sonigo *et al.*, 2019). Chemotherapy targets rapidly dividing cells, a hallmark of cancer, but this also makes healthy reproductive cells vulnerable to damage (Alimbetov *et al.*, 2018). The extent of chemotherapy’s impact on fertility depends on the individual’s ovarian reserve before treatment (Blumenfeld, 2012).

### Ovarian Follicles and Their Role in Female Fertility

Throughout a woman’s reproductive life, a small percentage of follicles are selected to mature and eventually release oocytes in a process known as ovulation (Johnson, 2015). However, most follicles never reach maturity and undergo a natural process called atresia, where they degrade and are reabsorbed by the body (Corriero *et al.*, 2021). This gradual depletion of follicles is a normal part of the aging process, but when accelerated by external factors such as chemotherapy, it can significantly impair fertility (Yan *et al.*, 2022). Each ovarian follicle contains an oocyte surrounded by support cells that help regulate its development and maturation (Clarke, 2018). The health of these oocytes is critical for reproductive success, and damage to them can lead to infertility or diminished fertility. Chemotherapy, particularly certain classes of drugs, can interfere with this process by damaging the DNA within oocytes or inducing apoptosis, reducing viable oocytes over time (Winship *et al.*, 2018).

### Chemotherapy and Its Mechanisms of Ovarian Damage

Chemotherapy drugs exert their effects by interfering with cell division and inducing DNA damage (Sun *et al.*, 2021). While this is crucial for killing cancer cells, it also impacts other rapidly dividing cells, including those in the ovaries. Among the most harmful chemotherapy agents for fertility are alkylating agents, which include drugs like cyclophosphamide, ifosfamide, and busulfan (Kapoor *et al.*, 2025). These drugs work by directly binding to the DNA in cells, creating cross-links and breaking DNA strands, which prevents cells from dividing and ultimately leads to cell death (Bergamo *et al.*, 2018). In ovarian follicles, this can destroy oocytes, causing a significant reduction in the ovarian reserve (Monniaux, 2018). The ovaries are particularly susceptible to chemotherapy-induced gonadotoxicity because they contain cells that are in various stages of division and development. Preantral and antral follicles, which are the follicles closest to releasing mature oocytes, are especially vulnerable to damage (Das *et al.*, 2023). These follicles require a precise hormonal environment and intact DNA to complete the maturation process, and chemotherapy can disrupt both, either by directly damaging the oocyte’s DNA or by impairing the support cells and hormone production needed for development (Winship *et al.*, 2018). One major risk posed by toxic elements is oxidative stress, which also occurs when chemotherapy drugs generate free radicals that can damage cellular structures, including DNA, proteins, and lipids (Cauli, 2021; Talaie *et al.*, 2025). Environmental factors such as air pollution, UV radiation, and chemical toxins induce oxidative stress by generating excess reactive oxygen species, which overwhelm antioxidant defenses and damage cellular components like lipids, proteins, and DNA (Alaee, 2018; Banagar *et al.*, 2018; Alaee *et al.*, 2024a; Khodabandeh *et al.*, 2025). Antioxidants neutralize reactive oxygen species (ROS) and protect cells from oxidative damage by donating electrons, scavenging free radicals, and enhancing endogenous defense systems (Patil *et al.*, 2015; Flora *et al.*, 2021; Neisy *et al.*, 2025; Zahedi *et al.*, 2025).

Oocytes, being long-lived cells, are particularly prone to accumulating damage from oxidative stress. This can lead to DNA mutations or other types of molecular damage that not only reduce the chances of successful fertilization but may also raise concerns about the quality and health of any offspring (Musson *et al.*, 2022).

### Long-Term Consequences: Premature Ovarian Failure and Diminished Ovarian Reserve

The most serious consequence of chemotherapy on female fertility is the potential for premature ovarian failure (POF), also known as primary ovarian insufficiency (POI) (Hao *et al.*, 2019). POF occurs when the ovaries stop functioning properly before the age of 40, leading to early menopause and a loss of fertility. The onset of POF is marked by the cessation of menstruation (amenorrhea), low levels of estrogen, and high levels of follicle-stimulating hormone (FSH), which indicate that the ovaries are no longer producing oocytes. POF can be a direct result of extensive damage to the ovarian reserve caused by chemotherapy. Since women are born with a limited number of oocytes, once these are depleted, the ovaries cannot regenerate them (Monniaux, 2018). The risk of POF is particularly high in women who receive high doses of alkylating agents or undergo prolonged chemotherapy regimens (Cui *et al.*, 2018). Women who experience POF may have no remaining viable oocytes, making natural conception impossible and requiring alternative methods like donor oocytes or adoption to achieve parenthood.

In cases where chemotherapy does not result in complete ovarian failure, it can still lead to diminished ovarian reserve (DOR) (Spears *et al.*, 2019). This condition is characterized by a reduction in the number of viable oocytes, which can make conception more difficult and reduce the chances of successful in vitro fertilization if fertility treatment is pursued (Parsanezhad *et al.*, 2017; Al Abdali & Gowri, 2021; Ashourzadeh *et al.*, 2024). Women with DOR often face shorter reproductive windows and may experience challenges with oocyte quality, which further complicates efforts to conceive (Swan & Colino, 2022).

### Hormonal Disruption and Its Impact on Reproduction

In addition to directly damaging oocytes, chemotherapy can disrupt the hormonal environment required for normal ovarian function (Xiong *et al.*, 2021). Ovarian follicles rely on the balanced production of several key hormones, including FSH and luteinizing hormone (LH), which stimulate the growth and maturation of oocytes (Salih & Jaafar, 2013; Ma *et al.*, 2023). Chemotherapy can interfere with the hypothalamic-pituitary-ovarian axis, disrupting the normal feedback loops that regulate these hormones (Markowska *et al.*, 2024). This disruption can lead to irregular menstrual cycles, anovulation, and ultimately, infertility (Song & Li, 2021). Hormonal imbalances can also contribute to temporary amenorrhea, folliculogenesis arrest, and disruption of oocyte maturation (Alaee *et al.*, 2024b; Singh *et al.*, 2024). In some women, chemotherapy-induced hormonal disruptions may persist, reducing their chances of conceiving naturally even if menstruation resumes (Markowska *et al.*, 2024).

### Factors Influencing Fertility Outcomes Post-Chemotherapy

Several factors determine whether chemotherapy will cause temporary or permanent infertility. One of the most significant factors is the age of the patient at the time of treatment (Blumenfeld, 2002). Younger women generally have a larger ovarian reserve, which offers some protection against complete ovarian failure (Richardson *et al.*, 2014). They are more likely to recover ovarian function after chemotherapy, though this is not guaranteed. Older women, particularly those approaching menopause, have fewer remaining oocytes, making them more vulnerable to permanent infertility (Qiao *et al.*, 2014). The risk of long-term fertility issues increases significantly for women over 35 who undergo chemotherapy (Blumenfeld, 2012). The type of chemotherapy drug used is another critical factor. Alkylating agents are among the most gonadotoxic, causing the greatest damage to the ovaries (Bhardwaj *et al.*, 2023). Other chemotherapy drugs, such as platinum-based compounds (e.g., cisplatin) and taxanes (e.g., paclitaxel), also pose a risk but may cause less severe damage depending on the dosage and duration of treatment (Rottenberg *et al.*, 2021). The overall dosage and length of chemotherapy also influence fertility outcomes (van den Berg *et al.*, 2021). Higher doses of chemotherapy, as well as prolonged treatment regimens, increase the likelihood of permanent ovarian damage (Mauri *et al.*, 2020). Additionally, combination therapies, which involve using multiple chemotherapy agents, are more likely to cause irreversible harm to the ovaries. Figure 1 shows the gonadotoxic effects of chemotherapy drug on female fertility.

**Figure 1:**
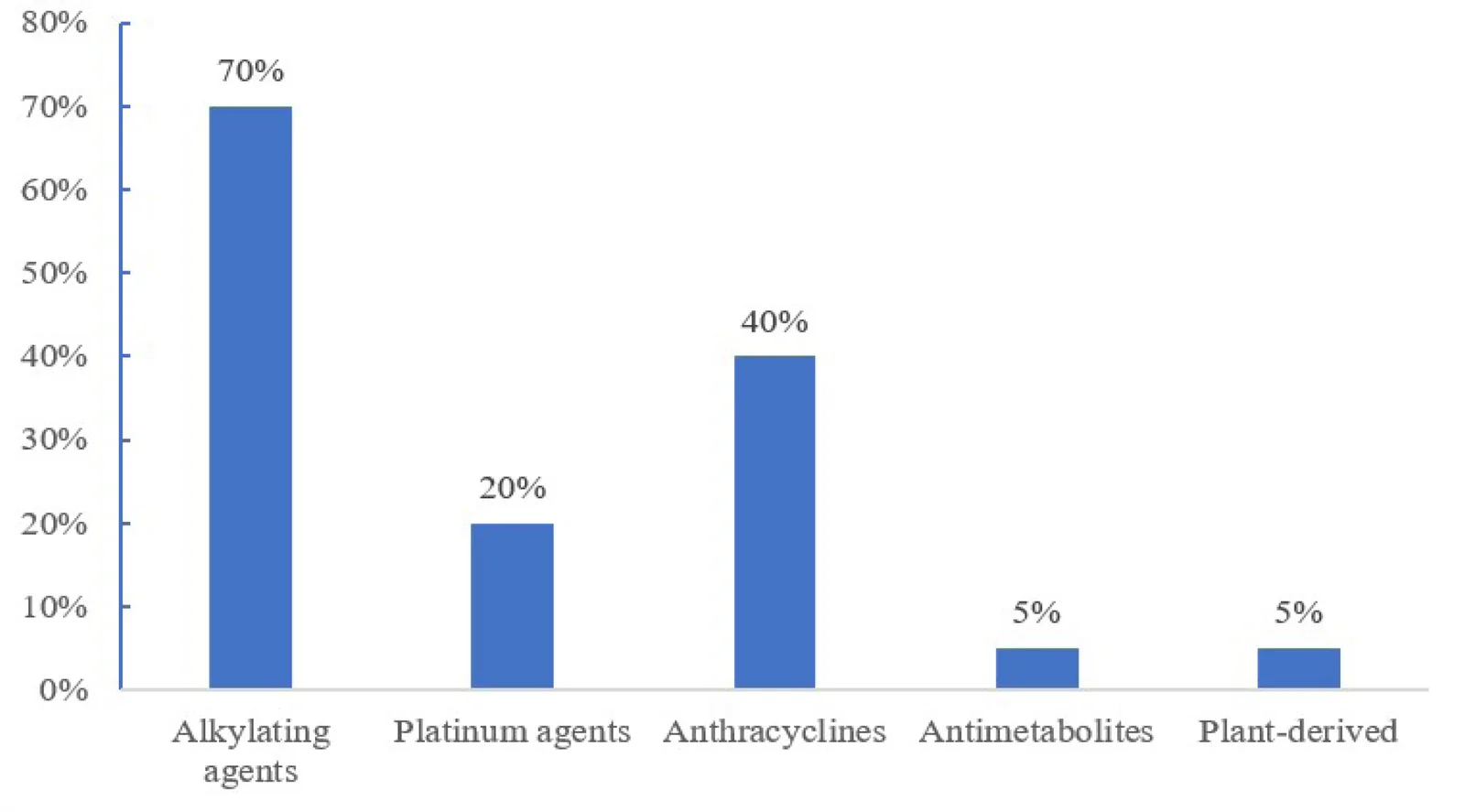
Comparative gonadotoxic effects of major chemotherapy drug classes on female fertility (data have been extracted from Mulder *et al.* (2021), Dumenigo *et al.* (2024), Himpe *et al.* (2023), and Santaballa *et al.* (2022).

## CHEMOTHERAPY’S EFFECTS ON MALE FERTILITY

Chemotherapy carries significant risks for male fertility (Di Tucci *et al.*, 2022). The primary target of chemotherapy in men is the process of spermatogenesis, which occurs in the testes and involves sperm production from precursor germ cells (Lopes *et al.*, 2021). These germ cells are among the most rapidly dividing cells in the body, making them particularly susceptible to the damaging effects of any toxicants (Mohammadi *et al.*, 2023). The drugs used in chemotherapy can lead to a reduction in sperm count, known as oligospermia, or even a complete absence of sperm in the semen, referred to as azoospermia (Mendes *et al.*, 2022). Additionally, some chemotherapy agents can damage the DNA within sperm, raising concerns about fertility and the potential for genetic abnormalities in future offspring (Meistrich, 2020).

### Mechanism of Action

The mechanism of chemotherapy affecting male fertility revolves around its impact on the rapidly dividing germ cells responsible for sperm production (Duffin *et al.*, 2024). Chemotherapy drugs, especially those classified as alkylating agents, can inflict substantial damage on these cells, leading to impaired spermatogenesis (Sriram *et al.*, 2024). By disrupting the normal cell division process, these drugs can interfere with the production of healthy sperm, causing various fertility issues. This damage is compounded by the fact that sperm production is a continuous process; therefore, any disruption can have immediate and long-lasting effects on a man’s fertility. The effects may not only result in reduced sperm counts but may also lead to sperm with compromised motility and morphology, further diminishing the chances of successful conception (Ben-Aharon *et al.*, 2010).

### Impact of Specific Chemotherapy Agents

The specific chemotherapy agents used play a critical role in determining the degree of fertility impairment in men (Figure 2) (Delessard *et al.*, 2020). Alkylating agents, such as cyclophosphamide and chlorambucil, are known for their high gonadotoxic potential (Sriram *et al.*, 2024). These drugs can cause long-term or even permanent infertility by damaging the testicular germ cells directly (Lorenzi *et al.*, 2021). The likelihood of developing infertility increases with the cumulative dose and duration of treatment. Platinum compounds, including cisplatin, also pose a significant risk to male fertility (Tian En *et al.*, 2020). These drugs can harm the spermatogenic cells, leading to both temporary and, in some cases, permanent infertility (Schrader *et al.*, 2001). Men treated with these agents may experience varying degrees of fertility impairment based on their response to treatment. Other classes of chemotherapy drugs, such as antimetabolites and microtubule inhibitors (e.g., vinblastine and paclitaxel), have a moderate risk of inducing infertility (Markowska *et al.*, 2024). While many men may experience temporary infertility during and after treatment, some may regain normal sperm production within months. However, the timeline for recovery can vary significantly among individuals, with some men taking years to return to baseline sperm counts, if they recover at all.

**Figure 2:**
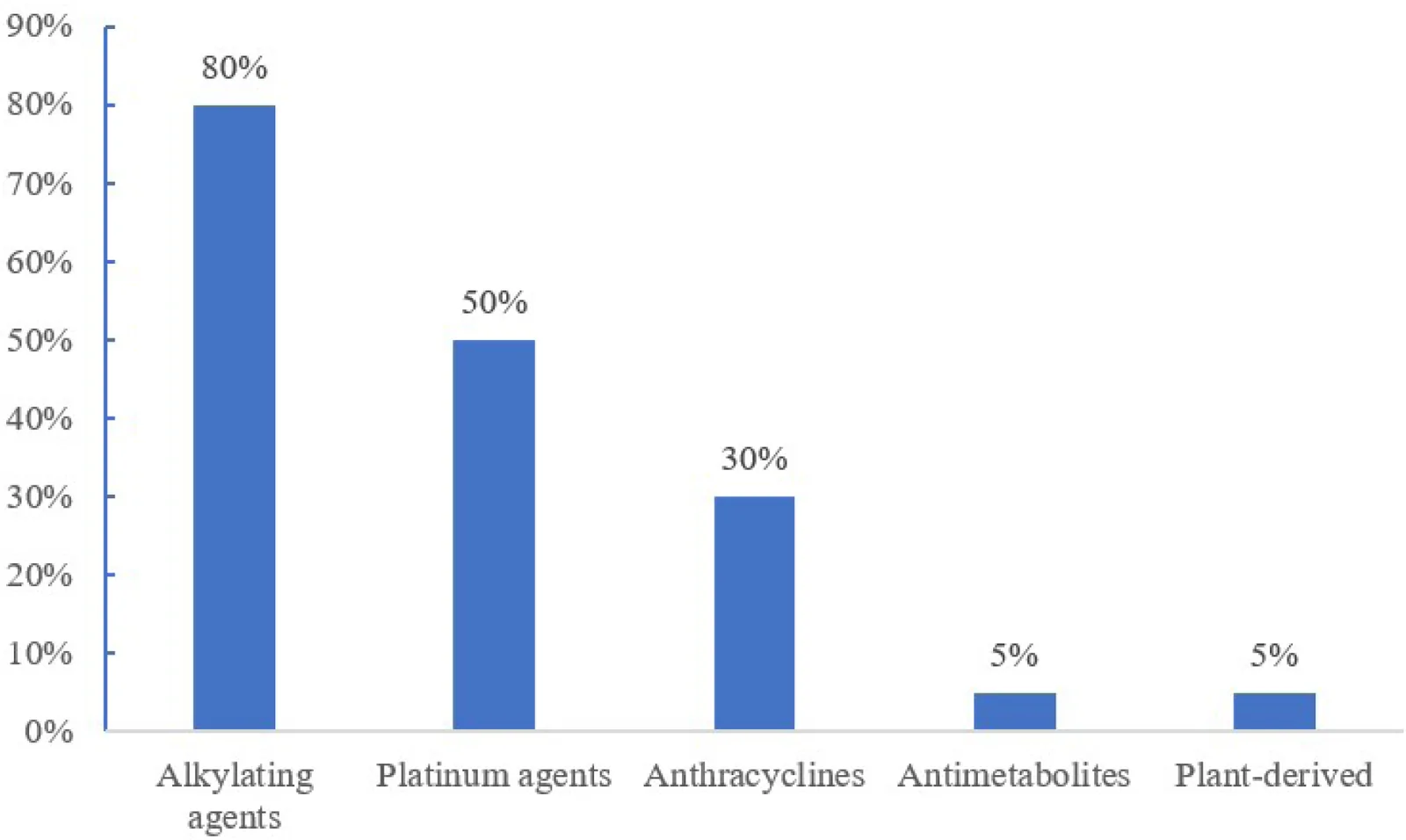
Comparative gonadotoxic effects of major chemotherapy drug classes on male fertility (the data have been extracted from Himpe et al., 2023; Reynolds & McKenzie, 2023).

### Other Reproductive Effects of Chemotherapy in Men

Beyond directly impacting sperm production, chemotherapy can induce other reproductive issues in men (Mendes *et al.*, 2022). One notable concern is hormonal disruption, particularly regarding the production of testosterone. Chemotherapy may affect the hormonal signaling pathways that regulate testosterone synthesis, potentially impacting sexual function, libido, and overall reproductive health (Babaniyi *et al.*, 2024). While hormonal disruptions are less common than the direct effects on sperm, they can still contribute to fertility challenges and affect a man’s quality of life. Additionally, testicular damage can occur as a result of chemotherapy (Amiri *et al.*, 2021). The structural integrity of the testicles is crucial for proper sperm production, and any damage to the testes can further impair fertility. The cumulative effect of chemotherapy on both sperm production and testicular health underscores the importance of monitoring reproductive health in men undergoing cancer treatment (McHugh *et al.*, 2024).

## SHORT-TERM *VS*. LONG-TERM EFFECTS

The effects of chemotherapy on male fertility can be categorized into short-term and long-term outcomes (Yumura *et al.*, 2023). In the short term, many men experience a temporary reduction in sperm count, motility, or quality during chemotherapy (Yumura *et al.*, 2023). This transient infertility can be distressing, especially for men who are planning to start families. While sperm production often resumes after the completion of chemotherapy, the timeline for recovery is unpredictable (Schlegel *et al.*, 2021). In some cases, it can take several months to years for sperm counts to return to normal levels, and not all men will fully recover their fertility. In contrast, the long-term effects of chemotherapy may include permanent infertility, especially in men who receive high doses of gonadotoxic drugs (Duffin *et al.*, 2024). Older men are particularly vulnerable; their reproductive function may already be declining due to age, and chemotherapy may exacerbate this decline, leading to an inability to produce viable sperm after treatment (Nguyen-Powanda & Robaire, 2020). The risk of long-term infertility necessitates careful consideration and planning for men undergoing chemotherapy who wish to preserve their reproductive options (Di Tucci *et al.*, 2022). Table 2 highlights that chemotherapy can cause temporary fertility issues, like amenorrhea in women and low sperm counts in men, which often improve in younger patients. However, long-term effects, such as permanent infertility from ovarian failure or azoospermia, are more likely with high doses of drugs like cyclophosphamide, especially in older individuals. These differences emphasize the need for early fertility planning to address both short-term setbacks and lasting impacts.

**Table 2:** Risk factors for chemotherapy-induced infertility.

Factor	Effect on Women	Effect on Men	Key Examples/Notes	Reference
Type of Drug	Alkylating agents (e.g., cyclophosphamide) can destroy oocytes, causing permanent infertility. Platinum drugs (e.g., cisplatin) also harm ovaries.	Alkylating agents (e.g., cyclophosphamide) often lead to no sperm (azoospermia). Platinum drugs (e.g., cisplatin) damage sperm production.	Cyclophosphamide, cisplatin are high-risk; taxanes are milder.	Bhardwaj *et al.*, 2023
Dosage	Higher doses increase the chance of losing all oocytes, leading to early menopause.	Bigger doses raise the risk of permanent sperm loss, especially with multiple drugs.	More damage with higher cumulative doses.	Poorvu *et al.*, 2019
Age	Older women (over 35) lose fertility faster due to fewer oocytes left, while younger women may recover better.	Older men (over 40) recover sperm slower or not; younger men have a better chance.	Age 35+ for women, 40+ for men heightens risk.	Horan, 2022
Pre-existing Fertility Health	Women with issues like endometriosis or PCOS lose oocytes more easily, risking infertility sooner.	Men with low sperm count or poor motility before treatment face worse fertility loss.	Conditions like PCOS or low sperm count worsen outcomes.	Papathanasiou *et al.*, 2024

Table 3 summarizes the shortand long-term effects of chemotherapy on male and female fertility, while Figure 3 illustrates the underlying mechanism of chemotherapy-induced infertility.

**Table 3:** Short-term vs. long-term effects of chemotherapy on fertility.

Time Frame	Effects on Women	Effects on Men	Recovery Potential	Influencing Factors	Reference
Short-term	Temporary amenorrhea (no periods) and hormonal changes during treatment.	Temporary low sperm count (oligospermia) or no sperm (azoospermia) during treatment.	High, especially in younger patients; often reverses within months to a year.	Age, milder drugs (e.g., taxanes).	Hao *et al.*, 2019
Long-term	Premature ovarian failure (POF) or diminished ovarian reserve (DOR), leading to permanent infertility.	Permanent azoospermia or reduced sperm quality, causing lasting infertility.	Low with high-dose gonadotoxic drugs; partial recovery possible in some.	High doses, alkylating agents (e.g., cyclophosphamide), older age.	Yumura *et al.* 2023

**Figure 3:**
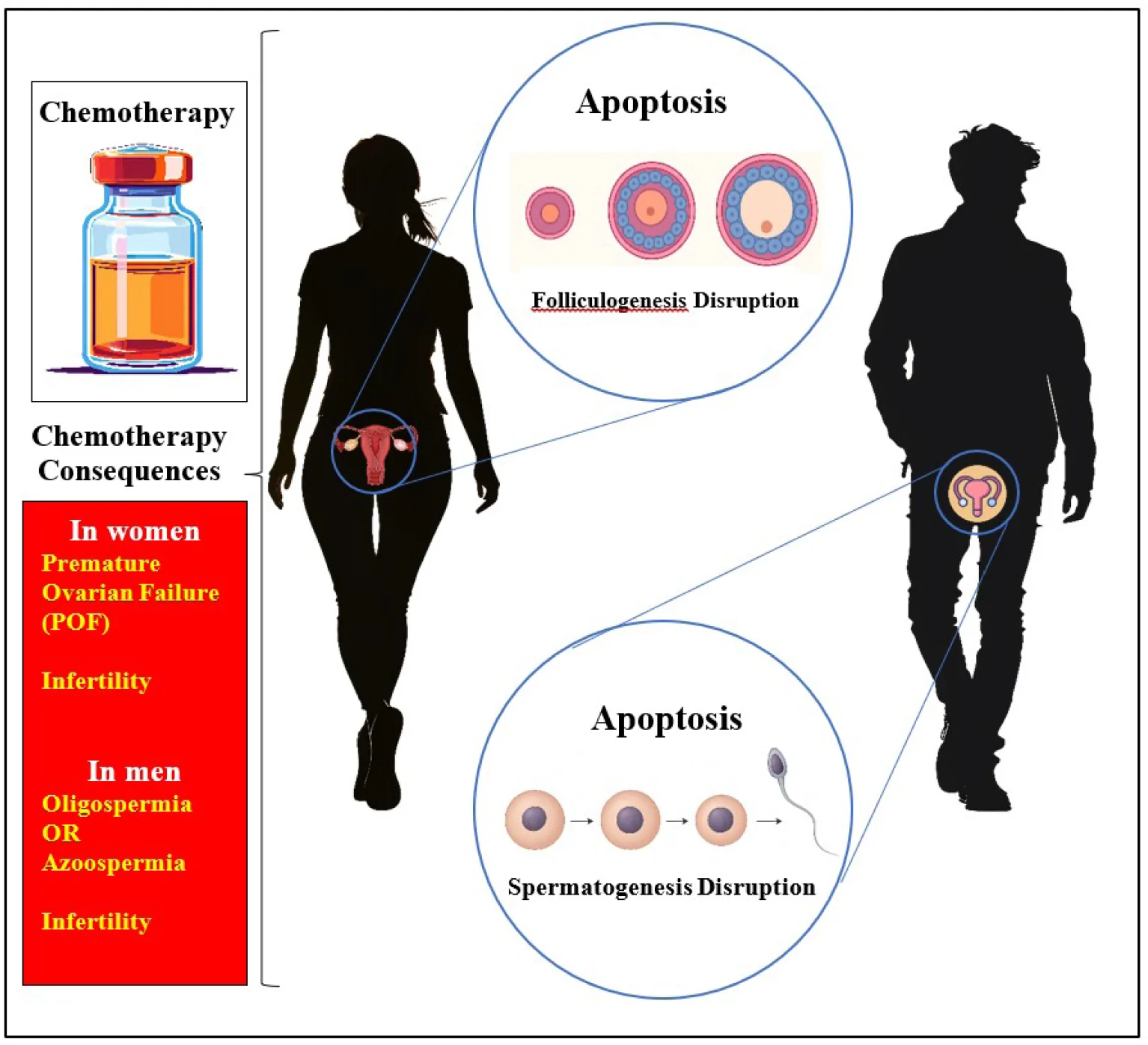
Schematic representation of folliculogenesis and spermatogenesis disruption after chemotherapy.

## FACTORS INFLUENCING FERTILITY LOSS IN CHEMOTHERAPY

Chemotherapy-induced infertility is influenced by several critical factors, each playing a significant role in determining the extent of reproductive damage and the likelihood of fertility recovery after treatment (Bhardwaj *et al.*, 2023). These factors include the type and dosage of chemotherapy drugs used, the age of the patient, and the individual’s pre-existing fertility health. Understanding these factors is essential for assessing the risk of infertility and considering fertility preservation options for cancer patients undergoing chemotherapy. Table 4 illustrates that chemotherapy-induced infertility hinges on drug type, dosage, age, and pre-existing fertility health. Alkylating agents and platinum compounds, especially at high doses, pose the greatest threat to reproductive cells in both women and men, often causing irreversible damage. Older patients face higher risks due to naturally declining fertility, while those with prior reproductive issues are more vulnerable. These factors highlight the need for tailored treatment plans and early fertility discussions to mitigate risks and preserve future reproductive options.

**Table 4:** Comparison of fertility preservation options for women and men.

Preservation Method	Target Population	Procedure Overview	Advantages	Limitations	Status	Reference
Oocyte Freezing	Adult Women	Ovarian stimulation with hormones, oocyte retrieval via minor surgery, freezing unfertilized oocytes	Well-established, high success with IVF, widely available	Requires 2-3 weeks, costly, not feasible if treatment is urgent	Established	Teo *et al.* (2022)
Embryo Freezing	Adult Women	Ovarian stimulation, oocyte retrieval, fertilization with sperm (partner or donor), freezing embryos	High success rate with IVF, suitable for couples, long storage viability	Requires a partner or donor sperm, time-intensive, ethical concerns for some	Established	Teo *et al.* (2022)
Ovarian Tissue Freezing	Prepubescent Girls, Adult Women (if urgent)	Surgical removal of ovarian tissue containing immature oocytes, freezing, future reimplantation	No hormonal stimulation needed, option for young girls, rapid procedure	Experimental, limited availability, risk of reintroducing cancer cells	Experimental	Jensen *et al.* (2022)
Ovarian Suppression	Adult Women	Use of GnRH agonists to temporarily suppress ovarian function during chemotherapy	Non-invasive, may reduce ovarian damage, adjunct to other methods	Uncertain efficacy, still under research, not a standalone solution	Experimental	Blumenfeld (2019)
Sperm Banking	Adult Men	Collection of semen samples via masturbation, freezing for future use in ART (e.g., IVF, ICSI)	Simple, cost-effective, high success rate, widely available	Requires viable sperm pre-treatment, not an option for azoospermic men	Established	Grin *et al.* (2021)
Testicular Tissue Freezing	Prepubescent Boys, Adult Men (if no sperm)	Surgical removal of testicular tissue with spermatogonia, freezing for future use	Only option for prepubescent boys, potential for future sperm generation	Experimental, requires future tech advances, limited clinical use	Experimental	Rives-Feraille *et al.* (2022)

### Type and Dose of Chemotherapy

One of the most influential factors in chemotherapy-induced fertility loss is the type and dosage of the chemotherapy drugs administered (Di Tucci *et al.*, 2022). Different chemotherapy agents vary in their level of gonadotoxicity, or their ability to damage reproductive cells. Alkylating agents, such as cyclophosphamide, ifosfamide, and busulfan, are among the most harmful to both male and female reproductive cells (Olowe *et al.*, 2024). These drugs work by binding to and cross-linking DNA, which disrupts cell division and leads to cell death. In reproductive cells, this process can result in the destruction of oocytes in women and sperm-producing germ cells in men, often leading to irreversible infertility. Cisplatin, a platinum-based compound, also poses a high risk of fertility loss. While it is effective in targeting cancer cells, cisplatin is highly toxic to germ cells, potentially causing long-term or permanent infertility, especially when administered at high doses (Katanić Stanković *et al.*, 2023). The cumulative dose of chemotherapy is a crucial factor; the higher the dose and the longer the treatment duration, the greater the risk of significant reproductive damage (van den Boogaard *et al.*, 2022). For some patients, even moderate doses of gonadotoxic drugs can lead to a reduction in ovarian reserve or sperm count, making it essential to tailor chemotherapy regimens carefully for patients concerned about fertility (Table 2).

### Age of the Patient

The age of the patient is another critical determinant of fertility loss during chemotherapy, particularly for women (Lee *et al.*, 2011). Female fertility is closely linked to the size and health of the ovarian reserve, which consists of a finite number of oocytes present from birth (Gleicher *et al.*, 2011). As women age, their ovarian reserve naturally declines, with the quantity and quality of oocytes diminishing over time. Older women, especially those approaching menopause, are at higher risk of permanent infertility following chemotherapy, as they may have fewer viable oocytes remaining before treatment even begins (Sparidaens *et al.*, 2022). The ovaries of younger women tend to be more resilient and may have a greater capacity to recover after chemotherapy, although this is not guaranteed. In men, the impact of age on chemotherapy-induced infertility is somewhat less pronounced but still important. Older men may experience a slower or incomplete recovery of sperm production following chemotherapy due to age-related declines in reproductive function (de Almeida *et al.*, 2024). As a result, men over the age of 40 undergoing chemotherapy may face a higher risk of permanent fertility loss than younger men. Both male and female patients should consider their age when assessing the potential impact of chemotherapy on their long-term fertility.

### Pre-existing Fertility Health

A patient’s pre-existing fertility health is a significant factor in determining their susceptibility to chemotherapy-induced infertility (Mauri *et al.*, 2020). Individuals with underlying fertility issues, such as endometriosis, polycystic ovarian syndrome (PCOS), or low sperm count, may be more vulnerable to the damaging effects of chemotherapy (Brassard *et al.*, 2008; Shiravani *et al.*, 2024). For women with conditions like endometriosis, where the health of the ovarian reserve may already be compromised, chemotherapy can further reduce the number of viable oocytes, increasing the likelihood of premature ovarian failure or diminished ovarian reserve (Mustapha *et al.*, 2021). Similarly, men with suboptimal sperm parameters, such as low sperm count or poor sperm motility, may experience a more profound impact on their fertility, as chemotherapy could exacerbate these issues (Blumenfeld, 2012). Patients with compromised reproductive health should be particularly cautious when undergoing chemotherapy and may benefit from pre-treatment fertility assessments (Di Tucci *et al.*, 2022). These evaluations can help determine baseline fertility and guide decisions about fertility preservation strategies, such as sperm banking for men or oocyte/embryo freezing for women. By understanding their unique reproductive risks, patients can make informed choices about safeguarding their fertility before beginning cancer treatment. The factors influencing fertility loss during chemotherapy are multifaceted and include the type and dose of chemotherapy drugs, the age of the patient, and their pre-existing fertility health. Alkylating agents and platinum compounds pose the highest risk of reproductive damage, particularly at high doses (Tian En *et al.*, 2020; Sriram *et al.*, 2024). Recognizing these factors is essential for assessing fertility risks and exploring preservation options to protect future reproductive potential.

## FERTILITY PRESERVATION OPTIONS FOR INDIVIDUALS UNDERGOING CHEMOTHERAPY

Fertility preservation strategies are available for patients who wish to have biological children after chemotherapy.

For patients undergoing chemotherapy who are concerned about their future reproductive potential, there are several medical interventions available to help preserve fertility. These options vary depending on the patient’s sex, age, and specific circumstances, such as whether they are preor post-pubertal. Fertility preservation methods are critical in helping patients retain the possibility of having biological children after cancer treatment (Klipstein *et al.*, 2020). Table 4 shows that fertility preservation options vary widely for women and men facing chemotherapy. These options highlight the importance of personalized planning to protect reproductive potential before cancer treatment begins.

### Fertility Preservation Options for Women Undergoing Chemotherapy

Fortunately, several fertility preservation strategies are available for women undergoing chemotherapy (Razeti *et al.*, 2023). The effectiveness and suitability of each depend on factors like age, ovarian reserve, and the type of chemotherapy being administered. Oocyte cryopreservation is one of the most common methods (Cho *et al.*, 2020). This involves stimulating the ovaries with hormones to produce multiple oocytes, which are then retrieved and frozen for future use. This technique allows women to preserve their oocytes before undergoing chemotherapy, providing an opportunity to conceive through IVF later (11). Another option is embryo freezing, which is similar to oocyte freezing but involves fertilizing the retrieved oocytes with sperm to create embryos before freezing them (Cho *et al.*, 2020). This approach is typically chosen by women who have a partner or wish to use donor sperm. Ovarian tissue cryopreservation is an emerging experimental technique where ovarian tissue is removed, frozen, and reimplanted after treatment to restore ovarian function (Eijkenboom *et al.*, 2022). This method is particularly useful for prepubescent girls who cannot undergo oocyte retrieval but still wish to preserve fertility. A significant concern with ovarian tissue transplantation is the potential reintroduction of malignant cells. To mitigate this risk, alternative approaches have been developed. Following ovarian tissue cryopreservation, follicles can be isolated and cultured in vitro, enabling their maturation without the need for tissue retransplantation. This technique eliminates the risk of cancer cell transfer while preserving fertility potential (Telfer *et al.*, 2023). In vitro maturation of oocytes from unstimulated or minimally stimulated ovaries offers another promising approach, particularly for patients who cannot undergo conventional ovarian stimulation.

New frontier approaches, including artificial ovary constructs using biomaterials, 3D scaffolds, and ovary-on-a-chip systems, are being explored to restore ovarian function and growth of follicles ex vivo (Dadashzadeh *et al.*, 2021).

Tissue engineering strategies using decellularized ovarian scaffolds are being developed to create a supportive microenvironment for follicular development while eliminating cancer cells (Alaee *et al.*, 2021; 2024b). These acellular matrices, when repopulated with patient-derived follicles or ovarian cells, may restore endocrine function and fertility without the risk of cancer recurrence (Dadashzadeh *et al.*, 2021). Other scaffold-based approaches incorporating biomaterials like alginate or fibrin aim to mimic the ovarian extracellular matrix to support follicle survival and growth (Dadashzadeh *et al.*, 2021). These emerging technologies represent important alternatives to traditional transplantation of cryopreserved ovarian tissue, particularly for patients with blood-borne malignancies where the risk of malignant cell transfer remains a significant concern (Kuchakzadeh *et al.*, 2024).

For some women, the use of GnRH agonists (gonadotropin-releasing hormone agonists) during chemotherapy may help protect the ovaries by temporarily suppressing ovarian function (Lee & Choi, 2021). By shutting down the ovaries and reducing hormonal stimulation, GnRH agonists aim to shield oocytes from chemotherapy’s damaging effects. However, the effectiveness of this approach is still under investigation and may not provide complete protection against fertility loss (Lee & Choi, 2021).

### Fertility Preservation Options for Men

Men undergoing chemotherapy also have a range of fertility preservation options, with sperm banking being the most common and well-established technique (Jensen *et al.*, 2022).

The most common fertility preservation method for men is sperm banking, which involves collecting and freezing sperm samples before starting chemotherapy (Liu *et al.*, 2021). The sperm can be stored for extended periods and used later in assisted reproductive technologies, such as in vitro fertilization or intracytoplasmic sperm injection (Moungala, 2023). Sperm cryopreservation is a straightforward and effective option for men, with success rates for conception remaining high even after long-term storage (Akbarzadeh-Jahromi *et al.*, 2022; Moungala, 2022). For men who can produce sperm, this method is highly recommended before undergoing chemotherapy, as sperm production may be impaired or lost permanently after treatment (Sharma & Sharma, 2020).

For prepubescent boys who have not yet begun producing sperm, traditional sperm banking is not an option. In such cases, an experimental technique known as testicular tissue freezing may be considered (Saulnier *et al.*, 2021). This procedure involves surgically removing a small portion of testicular tissue that contains sperm precursor cells, which are immature cells that have the potential to develop into mature sperm eventually (Mitchell & Ives, 2024). The tissue is then frozen and stored for future use, with the hope that advances in reproductive technology will allow these precursor cells to be used to restore fertility or generate viable sperm in the future. While this method is still in the experimental stages, it offers hope for young boys who would otherwise face a high risk of infertility due to cancer treatment (Mitchell & Ives, 2024).

Successful testicular tissue reconstruction for the reintroduction of cryopreserved spermatogonial stem cells is a critical step towards restoring fertility (Forouzandegan *et al.*, 2025). This process typically involves the enzymatic digestion of a sterile testicular cell suspension, which is then co-cultured with supportive somatic cells and often integrated into a three-dimensional biomaterial scaffold, such as decellularized testis, agarose, or Matrigel, to reform a tissue-like structure (Del Vento *et al.*, 2018). Following in vitro culture to allow for cell aggregation and reorganization, this reconstructed tissue is subsequently grafted, often subcutaneously or under the tunica albuginea, into an immunodeficient mouse host (Del Vento *et al.*, 2018). This in vivo environment provides the necessary physiological signals and vascularization to support the survival, engraftment, and crucially, the initiation of spermatogenesis from the transplanted spermatogonial stem cells, thereby validating the functional potential of the reconstructed testicular niche (Izadi *et al.*, 2025).

Improving the success of fertility preservation via sperm and oocyte cryopreservation requires a multi-pronged approach that addresses both logistical and biological challenges. A primary strategy is the urgent timeline optimization between diagnosis and the start of therapy to harvest gametes at the highest possible quality (Ozimic *et al.*, 2023). For post-pubertal males, this means multiple sperm collections if time permits, while for females, random-start ovarian stimulation protocols have revolutionized the process by allowing immediate initiation of cycle synchronization, shaving weeks off the timeline (Walker *et al.*, 2022). Biologically, the administration of antioxidants is a key intervention to counteract the oxidative stress induced by both the disease state and cancer treatments themselves. Supplementation with compounds like Coenzyme Q10, Melatonin, and Vitamin E can help protect mitochondrial function in oocytes and reduce DNA fragmentation in sperm, thereby improving post-thaw survival and developmental competence (Karimfar *et al.*, 2015; Cao *et al.*, 2022; Zhang *et al.*, 2024).

## POST-CHEMOTHERAPY FERTILITY ASSESSMENT AND TREATMENT

After chemotherapy, patients who wish to conceive may undergo fertility assessments to determine whether natural conception is possible. In cases where infertility is diagnosed, assisted reproductive technologies such as in vitro fertilization, sperm retrieval, or donor oocytes/sperm may be explored.

## PSYCHOLOGICAL AND EMOTIONAL IMPACT OF INFERTILITY

Infertility resulting from chemotherapy can have profound psychological and emotional consequences, particularly for young cancer survivors who have aspirations for future parenthood (Banser, 2022). Getting a cancer diagnosis can make people scared and unsure. Finding out they might not be able to have kids can make them feel even more sad, lost, and hopeless. Many people feel angry, sad, or worried when they realize they might not be able to have kids. For young people who beat cancer, not being able to have kids can affect more than just their feelings-it can also change things with their partner, family, and friends. The stress of trying to have a baby, along with the tough physical and emotional effects of cancer treatment, can put pressure on close relationships. Partners might not get the emotional side of not having kids, which can leave the person feeling alone. Doctors and nurses are really important in helping with these emotional struggles. They need to talk to patients about ways to save fertility and the chance of not having kids before starting chemo. These talks can help patients make smart choices about having kids later. Also, giving patients counseling or support groups can help them deal with their sadness and worries. Support groups can be particularly beneficial as they create a safe space for individuals to share their experiences and feelings with others who are facing similar challenges. These connections can help combat feelings of isolation and foster a sense of community. Mental health professionals trained in reproductive health can also offer therapeutic interventions tailored to the unique experiences of cancer survivors, helping them process their emotions and navigate the complexities of infertility (Banser, 2022; Dong *et al.*, 2023; Ensafdaran *et al.*, 2024). Overall, recognizing the psychological and emotional impact of infertility is vital in providing comprehensive care to cancer survivors. By addressing these issues, healthcare providers can help individuals regain a sense of control and hope for the future, whether that involves exploring alternative paths to parenthood or finding peace with their situation.

Low-income patients, rural residents, and those without health insurance often face significant barriers to timely fertility preservation, including high out-of-pocket costs, lack of nearby specialized clinics, and the need to travel long distances to access reproductive services (Peipert *et al.*, 2023). Studies from multiple countries have reported that patients from socioeconomically disadvantaged backgrounds are less likely to be referred to fertility specialists compared with those from higher-income groups (Flanagan *et al.*, 2022). Geographic disparities also play a major role, as rural or remote communities may have limited or no knowledge and access to reproductive endocrinologists or cryopreservation facilities, leading to delayed or missed opportunities for fertility preservation before cancer treatment (Letourneau *et al.*, 2012; Alaee *et al.*, 2019). In addition, cultural norms, language differences, and lack of awareness among patients and healthcare providers can further reduce the likelihood of timely referral and intervention (Letourneau *et al.*, 2012). Variations in national health policies and insurance coverage add another layer of inequality, with some healthcare systems fully covering fertility preservation costs, while others provide no reimbursement, making such (Gadson *et al.*, 2024).

## NANOPARTICLES’ EFFECTS ON HUMAN FERTILITY

The advent of nanoparticle-based cancer therapies represents a monumental leap forward in oncology, offering the potential for highly targeted drug delivery that maximizes tumor destruction while minimizing the systemic side effects that have long plagued conventional treatments like chemotherapy and radiation (Abbasi, 2017; Taghipour *et al.*, 2017). By engineering these microscopic carriers to seek out and penetrate cancerous cells specifically, physicians can administer powerful cytotoxic agents with unprecedented precision (Abdollahii *et al.*, 2020). This targeted approach spares much of the body’s healthy tissues from exposure, thereby reducing the severity of common side effects such as nausea, nerve damage, and extreme fatigue (Andone *et al.*, 2023). The promise of nanoparticles lies in their ability to make cancer treatment more effective and more tolerable, potentially turning certain cancers into manageable chronic conditions (Andone *et al.*, 2023).

However, this significant progress introduces a complex and serious secondary concern: the potential risk of infertility (Falchi *et al.*, 2018; Ilani *et al.*, 2018). While nanoparticles are designed for specificity, their small size and unique pharmacokinetic properties raise questions about unintended biodistribution. Studies have shown that some nanoparticles, particularly those with poorly optimized formulations, may impair spermatogenesis by causing oxidative stress and DNA damage (Ilani & Alaee, 2017; Falchi *et al.*, 2018). Nanoparticles can cross biological barriers, such as the blood-testis barrier, and accumulate in reproductive tissues, which can be harmful. Studies have shown that some nanoparticles, particularly those with poorly optimized formulations, may impair spermatogenesis by causing oxidative stress and DNA damage (Falchi *et al.*, 2018). This can lead to oligospermia, azoospermia, or abnormal sperm morphology, ultimately reducing male fertility (Sabetian *et al.*, 2022b). In women, the impact of nanoparticles on ovarian function and oocyte quality is less well understood, but concerns have been raised about their potential to disrupt the ovarian environment or harm oocytes. While nanoparticles offer groundbreaking potential in cancer treatment and even in diagnosing and treating infertility, their use must be carefully optimized to avoid harming reproductive health.

Researchers are working on refining nanoparticle formulations, incorporating antioxidants, vitamins, or other protective agents to reduce toxicity (Sabetian *et al.*, 2021).

This improved detection helps in the early diagnosis of both male and female infertility, leading to more timely and effective treatments. In therapeutic applications, nanoparticles can be designed to deliver targeted drugs to tissues (Taghipour *et al.*, 2017). For example, gold, zinc, silver, and cerium nanoparticles are under investigation for their ability to enhance sperm quality or protect oocytes from oxidative damage during assisted reproductive technologies, but some of them have adverse effects (Davis *et al.*, 2024).

Consequently, the development and deployment of these innovative treatments must be paired with proactive fertility preservation strategies. For patients of reproductive age, this means oncologists must integrate discussions about infertility risks into the informed consent process before starting treatment. The scientific community is also obligated to conduct rigorous long-term toxicology studies focused specifically on the gonadal impact of various nanoparticle formulations. Ultimately, the goal is to harness the life-saving power of nanotechnology without sacrificing the possibility of future life, ensuring that beating cancer does not come at the cost of a patient’s ability to build a family.

## CONCLUSION

Chemotherapy is a vital tool in combating cancer, yet its impact on fertility represents a significant challenge for patients of reproductive age. This review highlights how chemotherapy, particularly through alkylating agent compounds, disrupts ovarian function and spermatogenesis, often leading to diminished fertility or permanent infertility. The risk is modulated by factors such as drug type, dosage, patient age, and baseline fertility, with younger patients showing greater potential for recovery. Advances in fertility preservation, including oocyte, embryo, and sperm cryopreservation, alongside experimental techniques like ovarian and testicular tissue freezing, provide viable options for safeguarding reproductive potential. However, the emotional toll of infertility underscores the importance of pre-treatment counseling and psychological support. Ultimately, a multidisciplinary approach-integrating oncology, reproductive medicine, and mental health support-is essential to address the complex interplay between cancer treatment and fertility, empowering patients to make informed decisions about their reproductive futures.
